# The Web-Based Physician is Ready to See You: A Nationwide Cross-Sectional Survey of Physicians Using a Mobile Medical App to Evaluate Patients With Sexually Transmitted Diseases in China

**DOI:** 10.2196/10531

**Published:** 2018-10-30

**Authors:** Bolin Cao, Peipei Zhao, Cedric Bien-Gund, Weiming Tang, Jason J Ong, Thomas Fitzpatrick, Joseph D Tucker, Zhenzhou Luo

**Affiliations:** 1 Shenzhen University Shenzhen China; 2 University of North Carolina Project - China Guangzhou China; 3 Social Entrepreneurship to Spur Health Guangzhou China; 4 Shenzhen Nanshan Center for Chronic Disease Control Shenzhen China; 5 University of North Carolina at Chapel Hill Chapel Hill, NC United States; 6 London School of Hygiene and Tropical Medicine London United Kingdom

**Keywords:** STD, physician, mobile app, China, mobile phone, mHealth

## Abstract

**Background:**

Web-based medical service provision is increasingly becoming common. However, it remains unclear how physicians are responding to this trend and how Web-based and offline medical services are linked.

**Objective:**

The objectives of this study were to examine physicians’ use of mobile medical apps for sexually transmitted disease (STD) consultations and identify the physicians who frequently use mobile medical apps to evaluate patients with STD.

**Methods:**

In August 2017, we conducted a nationwide cross-sectional survey among physicians registered on a mobile medical app in China. We collected data on physicians’ demographic information, institutional information, and Web-based medical practices. We compared physicians who used mobile medical apps to evaluate patients with STD frequently (at least once a week) with infrequent users. Bivariate and multivariate logistic regressions were used to identify physicians who frequently evaluated patients with STD on mobile medical apps.

**Results:**

A total of 501 physicians participated in the survey. Among them, three-quarters were men and the average age was 37.6 (SD 8.2) years. Nearly all physicians (492/501, 98.2%) recommended their last Web-based patient with STD to subsequently see a physician in the clinic. More than half (275/501, 54.9%) of physicians recommended STD testing to Web-based patients, and 43.9% (220/501) provided treatment advice to patients with STD. Of all physicians, 21.6% (108/501) used mobile medical apps to evaluate patients with STD through Web more than once a week. Overall, 85.2% (427/501) physicians conducted follow-up consultation for patients with STD using mobile medical apps. Physicians working at institutions with STD prevention materials were associated with frequent evaluation of patients with STD on mobile medical apps (adjusted odds ratio=2.10, 95% CI 1.18-3.74).

**Conclusions:**

Physicians use mobile medical apps to provide a range of services, including Web-based pre- and posttreatment consultations and linkage to offline clinical services. The high rates of referral to clinics suggest that mobile medical apps are used to promote clinic-seeking, and not replace it. Physicians’ use of mobile medical apps could benefit sexual minorities and others who avoid formal clinic-based services.

## Introduction

In recent years, the growth of mobile health (mHealth) technology has created new ways for physicians to evaluate and follow-up patients. Physicians now use smartphone mobile medical apps to counsel patients, recommend testing, write prescriptions, and monitor them after clinical visits [1,2]. In addition, mobile medical apps provide physicians a tool to communicate with patients, contact other physicians in their network, follow trends in their specialties, and streamline office workflow [3,4]. Globally, mobile medical apps are an emerging technology [5], with physician users in developed (eg, the United States and the United Kingdom) [6-8] and developing countries (eg, Turkey and rural South Africa) [9,10].

Despite the proliferation of omnibus usage, mobile medical apps may particularly appeal to male patients seeking care for sexually transmitted diseases (STDs). Young men are less likely than their female counterparts to discuss sexual health, STD risk reduction, and relationships during routine clinic visits [11]. Patients seeking STD care are often reluctant to seek health care due to concerns about inhospitable clinical environments and perceived lack of confidentiality [12,13]. Meanwhile, Web-based environments provide relative convenience, low price, and anonymity for patients compared with seeing a physician in-person [13,14].

Smartphone use is rising rapidly around the world, especially in China and other low- and middle-income countries. In China, smartphone ownership among adults aged 18-34 years reached 93% in 2015 [15]. In addition, mobile medical apps are rapidly developing, with substantial growth rates each year [16]. However, little is known about physicians’ use of mobile medical apps for communicating with patients in China. Mobile medical apps have the potential to facilitate physician-patient interactions and promote linkage to care, that is, Web-based patients with an STD who receive in-person treatment [17,18]. The functions of mobile medical apps may include the following: appointment-making (free); medical education and scholarly articles (free); and telemedicine, such as Web-based pretreatment consultation and posttreatment consultation (fee for service) [16]. Mobile medical apps can allow patients living in remote areas to consult physicians in first-tier cities via phone, short message service (SMS) text message, or image. Furthermore, patients can save time from waiting in queues and traveling to facilities for in-person consultations.

In this study, we describe physicians who have evaluated male patients with STD through a mobile medical app in China. Physicians who frequently use mobile medical apps to evaluate patients might play crucial roles in expanding STD services as they could provide some knowledge and information to those patients who are concerned about inhospitable clinical environments and perceived lack of confidentiality. We aimed to examine physicians’ use of mobile medical apps, for example, physician reasons for using apps and the linkage between Web-based consultation and offline practice and also investigate factors associated with physicians’ frequent use of mobile medical apps.

## Methods

### Study Design, Setting, and Participants

We conducted a cross-sectional, nationwide Web-based survey from August 14 to August 23, 2017. We partnered with Xingren Doctor app (Xingren app), a popular mobile medical app in China, for participant recruitment (Multimedia Appendix 1). The app has >400,000 unique, verified physician users since its launch in 2014 and allows physicians to communicate with their patients remotely. Eligible physicians were those who practiced in specialties that had a higher chance of managing patients with STD, that is, dermatovenereology, urology, proctology, internal medicine, pediatrics, infectious disease, and general practice. We chose to select 500 physicians because of feasibility considerations and discussions with the physician app. A total of 120,126 physicians were registered in these specialties on the Xingren app. We distributed the participation link to physicians through the app messaging system, excluding users who had not logged into the app in the past 6 months. The whole distribution was conducted in 4 rounds. Randomization algorithms with RIGHT and RANDBETWEEN functions in Excel were used to select the identity numbers of physicians to distribute the survey link. After receiving the survey link, physicians who were interested in participation could click the survey link, which would direct them to a Web-based survey hosted on Sojump, a popular Web-based survey tool in China.

Eligible physicians included those who had seen at least 1 male patient with STD in the past 12 months. We aimed to exclude physicians who only evaluated female patients with STD (largely gynecologists), but who never managed any male patients with STD. We chose to focus on examining the last male patients with STD seen by physicians as we were particularly interested in physicians’ practice to ask about men’s sexual behavior, condom use, and history of STDs. Participants were asked to sign an electronic informed consent form before starting the survey. Eligible participants received a small financial incentive (~US $4.50) for their participation.

### Variables

Our survey instrument included domains of sociodemographic information, clinic information, clinical practice with male patients with STD, and use of mobile medical apps. The outcome variable was the binary variable of the frequency at which physicians saw patients with STD using mobile medical apps (more than once a week vs less than once a week). The exposure variables included physicians’ sociodemographic information, clinic information, and clinical practice with male patients with STD.

### Quantitative Variables

We assessed the frequency of using mobile medical apps to evaluate patients with STD among physicians as a binary outcome variable to compare physicians who saw patients with STD more or less than once a week (frequent users vs infrequent users) [19]. Physicians who use mobile medical apps to frequently evaluate patients at this stage tend to be early adopters of new mHealth care technologies [20]. Examining the early adoption behaviors can inform the further adoption among other STD physicians.

### Measures

Physicians’ sociodemographic characteristics included age, sex, education, and medical specialty. Physicians’ clinic information included the following data: type of affiliated medical institution, level of care (primary, secondary, or tertiary), type of hospital (public or private), availability of condoms and lubricants, and availability of STD prevention pamphlets. Physicians’ clinical practices with male patients with STD regarding obtaining sexual histories and addressing risk reduction were asked, such as whether they asked about patients’ sexual experience with other men or transgender individuals and whether they recommended patients to test for STDs (HIV and syphilis) [21].

Furthermore, physicians were asked about their use of mobile medical apps (including but not limited to the Xingren app) to provide medical services. All physicians were asked about their reasons for using mobile medical apps for consultations and how the Web-based advice service was connected with their offline clinical services.

Furthermore, we collected data on physicians’ Web-based consultation behaviors. We defined consultations as interactions that provided medical advice to patients either in real-time or in a delayed manner. We assessed physicians’ experience in whether they had used mobile medical apps to provide initial consultation or follow-up consultation through Web in the last 12 months. We assessed whether physicians recommended HIV or STD testing and provided treatment advice for a patient, based only on a Web-based encounter without seeing a physician in-person. Moreover, we asked whether physicians conducted any follow-up consultations through Web after an initial in-person clinical encounter. The questions about how the Web-based consultation services were linked with their offline medical services were also asked. The approaches of linkage included third-party medical platforms, the medical institutions’ services on WeChat (which is the most popular social media platform in China), the clinic’s self-developed Web-based physician-patient communication platform, or others.

In addition, we asked physicians to recall their use of mobile medical apps with their last Web-based patient with STD. We assessed the medium (ie, through SMS text message, image, audio, or video) that physicians used to assess their patient’s STD symptoms and the time physicians spent on the mobile medical apps to evaluate their patients. Besides, we asked physicians whether they felt more or less comfortable to ask the patients’ experiences on sexual behaviors, condom use, and history of STDs compared with during an in-person consultation. Physicians were asked whether they asked their patients to visit a hospital or a clinic in-person after the Web-based consultation. The survey was designed and reported following the Checklist for Reporting Results of Internet E-Surveys [22]. Overall, 15 individuals were pretested, and their feedback was incorporated into the final survey.

### Study Size

Overall, the survey link was distributed to 8098 physicians, and 1556 physicians opened the survey link. Among the 701 physicians who started the survey, 2 did not provide informed consent, 186 were excluded for having not seen at least 1 male patient with STD in the past 12 months, and another 12 surveys were invalidated due to a survey instrument error. Eventually, 501 physicians fulfilled eligibility criteria and completed the survey and were included in our data analysis.

### Ethics Statement

We obtained approval for this study from the ethics review committee to ensure participants’ confidentiality and anonymity at the Nanshan Center of Chronic Disease Control, Shenzhen, China (ll20170016) prior to survey launch.

### Statistical Analysis

Descriptive statistics were used to describe physicians’ sociodemographic information, medical institution information, medical practice experiences, and usage of mobile medical apps. We conducted bivariate and multivariate analyses using SPSS software (IBM SPSS Inc) to examine factors associated with physicians who saw patients with STD more than once a week. In addition, chi-square test and *t* test were used to compare the physicians who used mobile medical apps to evaluate patients with STD at least once a weak with those who did less than once a weak. Multivariable logistic regression models were adjusted for potential confounding variables, including age (continuous variable), sex, and education, as informed by previous studies [23,24].

## Results

### Physicians’ Personal, Institutional, and Behavioral Characteristics

The average age of the 501 physicians was 37.6 (SD 8.2) years, of whom 53.9% (270/501) were in the age range of 35-50 years. The majority of them (376/501, 75.0%) were men. Less than one-tenth (36/501, 7.2%) of physicians had an associate’s degree, 39.5% (198/501) had a bachelor’s degree, 43.1% (216/501) had a master’s degree, and 10.2% (51/501) had a PhD degree. The most common subspecialty was dermatovenerology (166/501, 33.1%), followed by urology (151/501, 30.1%), and general medicine (72/501, 14.4%).

**Table 1:** Personal, institutional, and behavioral characteristics of physicians who saw male patients with sexually transmitted diseases (STDs) in the past 12 months in China, 2017 (N=501).

Characteristics			Values
**Personal characteristics**			
	Age (years), mean (SD)		37.6 (8.2)
	**Age group (years), n (%)**		
		18-34	191 (38.1)
		35-50	270 (53.9)
		51-69	38 (7.6)
		>70	2 (0.4)
	**Sex, n (%)**		
		Male	376 (75.0)
		Female	125 (25.0)
	**Education, n (%)**		
		Associate degree	36 (7.2)
		Bachelor	198 (39.5)
		Master	216 (43.1)
		PhD	51 (10.2)
	**Specialty, n (%)**		
		Dermatovenerology	166 (33.1)
		Urology	151 (30.1)
		General medicine^a^	72 (14.4)
		Proctology	41 (8.2)
		Infectious Disease	34 (6.8)
		Others	37 (7.4)
**Institutional characteristics, n (%)**			
	**Level of care**		
		Primary	34 (6.8)
		Secondary	145 (28.9)
		Tertiary	322 (64.3)
	**Type of medical institute**		
		Public	449 (89.6)
		Private	52 (10.4)
	**Free condom and lubricants available**		
		Yes	260 (51.9)
		No	241 (48.1)
	**STD prevention pamphlets available**		
		Yes	377 (75.2)
		No	124 (24.8)
**Offline medical practice in the last 12 months, n (%)**			
	**Had seen men who have sex with men patients with STD**		
		Yes	267 (53.3)
		No	234 (46.7)
	**Had seen transgender patients with STD**		
		Yes	92 (18.4)
		No	409 (81.6)
	**Asked about condom use all the time**		
		Yes	136 (27.1)
		No	365 (72.9)
	**Recommended STD testing^b^ all the time**		
		Yes	211 (42.1)
		No	290 (57.9)

^a^General medicine includes internal medicine, general practice, and pediatrics.

^b^STD testing means HIV testing and syphilis testing.

Most physicians worked at tertiary care medical institutions (322/501, 64.3%), 28.9% (145/501) worked at secondary care medical institutions, and only 6.8% (34/501) worked at primary care medical institutions. The majority of physicians (449/501, 89.6%) worked at public hospitals and only 10.4% (52/501) worked in private hospitals. Nearly half (260/501, 51.9%) of the institutions where physicians worked provided free condom and lubricants to patients. Three-quarters (377/501, 75.2%) of individuals reported that their medical institutions had STD prevention pamphlets or educational materials.

Slightly more than half (267/501, 53.3%) of physicians had seen men who have sex with men (MSM) patients with STD and 18.4% (92/501) had seen transgender patients with STD in the last 12 months. Around one-quarter of physicians (136/501, 27.1%) asked about condom use all the time during patient encounters, and less than half of physicians (211/501, 42.1%) recommended STD testing for patients all the time in outpatient settings (Table 1).

### Physicians’ Usage of Mobile Medical Apps

Among all physicians, 6.2% (31/501) used mobile medical apps to evaluate patients with STD through Web several times a day, 2.8% (14/501) used once a day, 12.6% (63/501) used once a week, 21.2% (106/501) used once a month, 14.2% (71/501) used once every 3 months, and 42.9% (216/501) used less than once every 3 months. Overall, 21.6% (108/501) physicians used mobile medical apps to evaluate patients with STD through Web at least once a week (frequent users). The most commonly cited reason to adopt mobile medical apps was convenience (435/501, 86.8%), followed by usefulness (231/501, 58.1%), novelty (205/501, 40.9%), and extra money (155/501, 30.9%).

More than half of the physicians surveyed (275/501, 54.9%) recommended HIV or STD testing and 43.9% (220/501) provided treatment advice for patients with STD through Web via mobile medical apps in the past 12 months. Of all physicians, 85.2% (427/501) had seen a patient with STD in-person first and then had a Web-based follow-up conversation.

About one-quarter (127/501, 25.3%) of all physicians reported that their Web-based services were organized by the clinics where they saw patients with STD in-person. Of these, nearly half (55/127, 43.3%) of the Web-based services were implemented through third-party platforms. Furthermore, WeChat services (40/127, 31.5%), clinics’ Web-based physician-patient communication platforms (25/127, 19.7%), and others (7/127, 5.5%) were used.

Regarding physicians’ last encounter with male patients with STD on mobile medical apps, most patients (455/501, 90.8%) used SMS text message to describe symptoms, 77.0% (386/501) used an image, 30.5% (153/501) used audio, and 5.2% (26/501) used video. More than three-quarters (394/501, 78.6%) of patients used more than one medium of communication. The majority (432/501, 86.2%) of physicians felt more comfortable on mobile medical apps asking about sexual behaviors, condom use (429/501, 85.5%) and STD history (429/501, 85.5%) compared with asking in-person (Multimedia Appendix 2). Physicians, on average, spent 10 minutes (interquartile range: 5.5-20 minutes) to provide Web-based consultation or advice on treatment to the last seen male patient with STD. Nearly all (492/501, 92.8%) physicians recommended the last seen Web-based patient to visit clinics in-person after the Web-based consultation (Table 2).

### Correlates of Evaluating Male Patients With Sexually Transmitted Disease Using Mobile Medical Apps

The odds of being a frequent user of mobile medical apps was greater for those who worked at institutions with STD prevention materials (odds ratio [OR]=2.18, 95% CI 1.23-3.87) and those who had seen an MSM patient with STD in the last 12 months (OR=1.58, 95% CI 1.02-2.44). Multivariate analyses showed that physicians who worked at institutions with STD prevention materials (adjusted odds ratio [aOR]=2.10, 95% CI 1.18-3.74) and saw MSM patients with STD in the last 12 months (aOR=1.66, 95% CI 1.06-2.58) were associated with frequent evaluation of patients with STD more than once a week on mobile medical apps (Table 3).

**Table 2:** Usage of mobile medical apps among physicians who saw male patients with sexually transmitted diseases (STDs) in the past 12 months in China, 2017 (N=501).

Characteristics			Values
**General usage of mobile medical apps with patients with STD, n (%)**			
	**Frequency of using mobile medical apps to see male patients with STD**		
		At least once a week	108 (21.6)
		Less than once a week	393 (78.4)
	**Reasons to adopt mobile medical apps**		
		Convenience	435 (86.8)
		Usefulness	291 (58.1)
		Novelty	205 (40.9)
		Extra money	155 (30.9)
		Trendiness	96 (19.2)
		Others	17 (3.4)
	**Recommended STD testing for Web-based** **patients**		
		Yes	275 (54.9)
		No	226 (45.1)
	**Provided treatment advice for Web-based** **patients**		
		Yes	220 (43.9)
		No	281 (56.1)
	**Conducted follow-up consultation through Web**		
		Yes	427 (85.2)
		No	74 (14.8)
	**The clinic itself as an organizer of Web-based services**		
		Yes	127 (25.3)
		No	374 (74.7)
	**The linkage between Web-based and offline medical services**		
		Through the clinic’s WeChat page	40 (31.5)
		Through the clinic’s Web-based physician-patient communication platform	25 (19.7)
		Through a third-party platform	55 (43.3)
		Others	7 (5.5)
**Usage of mobile medical apps with the last seen male patient with STD**			
	**Media used by patients to describe symptoms, n (%)**		
		Short message service text message	455 (90.8)
		Image	386 (77.0)
		Audio	153 (30.5)
		Video	26 (5.2)
	**Felt more comfortable using mobile medical apps to ask about sexual behavior compared with in-person, n (%)**		
		Yes	432 (86.2)
		No	69 (13.8)
	**Felt more comfortable using mobile medical apps to ask about condom use compared with in-person, n (%)**		
		Yes	429 (85.5)
		No	72 (14.4)
	**Felt more comfortable using mobile medical apps to ask about STD experiences compared with in-person, n (%)**		
		Yes	429 (85.5)
		No	72 (14.4)
	Time spent on providing consultation or treatment to the patient (in minutes), mean (SD)		13.86 (SD 12.74)
	**Asked the patient to visit a hospital or clinic in-person, n (%)**		
		Yes	492 (98.2)
		No	9 (1.8)

**Table 3:** Factors associated with the frequency of using mobile medical apps to evaluate male patients with sexually transmitted diseases (STDs) among physicians in China, 2017 (N=501).

Characteristics		Using mobile medical apps to evaluate male patients with STD		*t* test or chi-squared test		*df* ^a^	Bivariate or multivariate logistical regression	
		At least once a week, n (%)	Less than once a week, n (%)				Odds ratio (95% CI)	Adjusted odds ratio (95% CI)
Age (years)		38.7 (8.1)^b^	37.25 (8.2)^b^	1.6^c^		499	1.02 (1.00-1.05)	N/A^d^
**Sex**								
	Male	86 (79.6)	290 (73.8)	1.5^e^		1	1.39 (0.83-2.33)	
	Female	22 (20.4)	103 (26.2)				Ref^f^	
**Education**								
	Associate degree	10 (9.3)	26 (6.6)	2.4^e^		3	2.07 (0.72-5.91)	
	Bachelor	46 (42.6)	125 (38.7)				1.63 (0.71-3.71)	
	Master	44 (40.7)	172 (43.8)				1.38 (0.60-3.14)	
	PhD	8 (7.4)	43 (10.9)				Ref	
**Specialty**								
	Dermatovenerology	54 (50.0)	112 (28.5)	30.5^e,g^		5	1.50 (0.66-3.40)	1.50 (0.65-3.46)
	Urology	34 (31.5)	117 (29.8)				0.90 (0.39-2.10)	0.74 (0.31-1.75)
	Proctology	6 (5.6)	35 (8.9)				0.53 (0.17-1.68)	0.47 (0.15-1.49)
	General medicine	2 (1.9)	70 (17.8)				0.09 (0.02-0.44)^h^	0.08 (0.07-0.39)^h^
	Infectious Diseases	3 (2.8)	31 (7.9)				0.30 (0.07-1.23)	0.28 (0.07-1.14)
	Others	9 (8.3)	28 (7.1)				Ref	Ref
**Level of care**								
	Primary	7 (6.5)	27 (6.9)	1.3^e^		2	1.03 (0.43-2.46)	0.86 (0.34-2.18)
	Secondary	36 (33.3)	109 (27.7)				1.31 (0.82-2.08)	1.19 (0.70-2.02)
	Tertiary	65 (60.2)	257 (65.4)				Ref	Ref
**Type of medical institute**								
	Public	98 (90.7)	351 (89.3)	0.2^e^		1	1.17 (0.57-2.42)	1.41 (0.65-3.06)
	Private	10 (9.3)	42 (10.7)				Ref	Ref
**Free condom and lubricants available**								
	Yes	63 (58.3)	197 (50.1)	2.3^e^		1	1.39 (0.91-2.14)	1.38 (0.89-2.13)
	No	45 (41.7)	196 (49.9)				Ref	Ref
**STD prevention pamphlets or educational materials available**								
	Yes	92 (85.2)	285 (72.5)	7.3^e,h^		1	2.18 (1.23-3.87)^h^	2.10 (1.18-3.74)^i^
	No	16 (14.8)	108 (27.5)				Ref	Ref
**Had seen men who have sex with men patients with STD in the last 12 months**								
	Yes	67 (62.0)	200 (50.9)	4.2^e,i^		1	1.58 (1.02-2.44)^i^	1.66 (1.06-2.58)^i^
	No	41 (38.0)	193 (49.1)				Ref	Ref
**Had seen transgender patients with STD in the last 12 months**								
	Yes	21 (19.4)	71 (17.1)	0.1^e^		1	1.10 (0.64-1.88)	1.08 (0.63-1.88)
	No	87 (80.6)	322 (77.2)				Ref	Ref
**Recommended STD testing for Web-based patients**								
	Yes	67 (62.0)	208 (52.9)	2.8^e^		1	1.45 (0.94-2.25)	1.37 (0.88-2.13)
	No	41 (38.0)	185 (47.1)				Ref	Ref
**Provided treatment advices for Web-based patients**								
	Yes	63 (58.3)	157 (39.9)	11.6^e,h^		1	2.10 (1.37-2.24)^g^	2.10 (1.36-3.25)^h^
	No	45 (41.7)	236 (60.1)				Ref	Ref
**Ever conducted follow-up consultation through Web**								
	Yes	104 (96.3)	323 (82.2)	13.4^e,g^		1	5.64 (2.01-15.81)^g^	5.93 (2.10-16.79)^g^
	No	4 (3.7)	70 (17.8)				Ref	Ref

^a^*df*: degrees of freedom.

^b^Mean (SD).

^c^*t* test.

^d^N/A: not applicable.

^e^Chi-squared test.

^f^Using mobile medical apps to evaluate patients with STD less than once a week as the reference group; Ref: reference group.

^g^*P*<.001.

^h^*P*<.01.

^i^*P*<.05.

## Discussion

### Principal Findings

Web-based medical services are becoming increasingly common [25]. We described how physicians in China use mobile medical apps to evaluate patients with STD and how Web-based consultations are linked to offline medical services, contributing to the emerging eHealth field from a physician’s perspective. This study expands on previous studies by examining physicians’ frequency of using mobile medical apps to evaluate patients, by exploring how Web-based recommendations correlate with in-person clinic visits, and by focusing on Web-based medical services.

Use of mobile medical apps by physicians to provide medical services is a growing trend. This study found a higher rate of physicians frequently using mobile medical apps to see patients with STD (108/501, 21.6%) compared with a study in Canada (14%) [26]. Chinese physicians are directly incentivized to provide services through the mobile app, receiving approximately US $3 (range, US $0-$30) per Web-based consultation [27]. A survey of urologists in the United States found that 28% used mobile medical apps for professional purposes [28]. Mobile medical apps allow physicians to interact with their patients, regardless of geography or time [29]. In addition, mobile medical apps allow patients to play a more active role in disease management [30]. However, in a survey of Chinese urologists, 76% of physicians complained that mobile medical apps took time away from clinical practice, and some mentioned that negative comments on Web diminished their enthusiasm for Web-based practice [24].

Physicians who have STD prevention materials available in their institutions were more likely to use mobile medical apps frequently to evaluate male patients with STD. Disease prevention materials are part of health promotion strategies to improve health literacy and access to care [31]. Carefully selected information and educational materials can have a positive effect on patients’ health literacy, risk behaviors, and, in turn, clinical outcomes [32]. In particular, STD prevention materials are aimed at successfully motivating behavioral change, knowledge, and attitudes [33]. When health education and communication materials are available in a medical institution, its physicians may be more interested in adopting new technologies to improve health care services.

We found that more than half of the surveyed physicians recommended STD testing. This is consistent with the Chinese STD guidelines that encourage prompt STD testing for at-risk individuals [34]. Studies have found that individuals who have sought STD information on Web might have higher STD risks [27]. Following this logic, those who have consulted about STDs on mobile medical apps might also have higher risks for STD; thus, recommending testing for STDs is necessary. In addition, studies in high-, middle-, and low-income countries have demonstrated that Web-based medical service experiences can promote STD testing [35,36]. A study in the United Kingdom found that offering internet-accessed STD testing increased test uptake and decreased time to get tested. However, it did not reduce time to treatment [37]. Physicians play a crucial role in patient counseling and recommending HIV or STD testing, and interactions with physicians have been correlated with increased HIV or STD test uptake among MSM [38].

Nearly all physicians in our survey recommended their Web-based patients to see a physician in-person after the Web-based consultation. This finding suggests that Web-based consultations can supplement, but do not replace, in-person clinical encounters [39,40]. This also indicates that mobile medical apps can facilitate the linkage of patients with STD to care and disease management. The convenience of using mobile medical apps may broaden clinical service access and provide a means of increasing initial care seeking, counseling, and support, particularly among sexual minorities or patients with STD who may not otherwise seek care [41]. Although a majority of physicians surveyed reported receiving SMS text messages and images from Web-based patients, Web-based encounters still provide less information compared with face-to-face encounters [42]. Inadequate information through Web-based consultation may lead to an inaccurate assessment or misdiagnosis, and physicians may, thus, be reluctant to rely on Web-based consultation alone [43]. Further research is needed to understand the benefits and risks associated with Web-based medical services. A systematic review of the clinical use of mobile medical apps, including video-based Skype, suggested that this approach is feasible for chronic disease management [44].

This study has practical and research implications. From a practice perspective, Web-based consultation can provide a supplementary channel for patients to receive medical advice. Mobile medical services may be particularly useful for patients with STD and sexual minorities who hesitate to seek formal clinic-based services. Future research should investigate how to improve mobile medical services and build patient-centered, physician-friendly platforms to facilitate physician-patient communication. In addition, future studies should investigate how best to implement mobile interventions targeting key populations.

### Limitations

This study has some limitations. First, the response rate for our Web-based survey (6.2%) was relatively low, but it was similar to other Web-based surveys among physicians (8.6%) [45]. Second, we conducted the survey using a mobile medical app platform, which may also result in selection bias of high-frequency mobile medical apps users. Although this study presented a common form of mobile medical apps, various mobile medical apps exist on the market [5]; thus, our findings should be transferred to other settings with caution. Third, the services provided by physicians using mobile medical apps were self-reported rather than based on the electronic records of their practice. Fourth, this study had a small proportion of physicians at lower-level clinical facilities. Nonetheless, this is consistent with the proportion of physicians in primary, secondary, and tertiary care settings who comprise roughly 10%, 30%, and 60% of all users, respectively, for the Xingren mobile app. Future studies can use other sampling methods to examine the patterns of physicians using mobile medical apps for clinical services.

### Conclusions

Mobile medical apps have been adopted by physicians to provide Web-based medical advice. Physicians’ use of mobile medical apps provides patients with STD with more opportunities to seek Web-based STD health services and link to offline services. Leveraging mobile medical apps to provide high-quality services for patients with STD symptoms may improve access to STD clinical care.

## Appendix

Multimedia Appendix 1 Flowchart of participants’ recruitment.

Multimedia Appendix 2 Physician experiences during the last online encounter with a patient with sexually transmitted disease (STD) in China, 2017.

## Abbreviations

*df*: degrees of freedom
mHealth: mobile health
MSM: men who have sex with men
SMS: short message service
STD: sexually transmitted disease
