# Supplementary figures and images for: The Web-Based Physician is Ready to See You: A Nationwide Cross-Sectional Survey of Physicians Using a Mobile Medical App to Evaluate Patients With Sexually Transmitted Diseases in China
Source: JMIR Mhealth Uhealth. 2018 Oct 30;6(10):e10531. doi: 10.2196/10531 (PMC6234337; doi:10.2196/10531)

**Multimedia Appendix 1.** Flowchart of participants' recruitment.

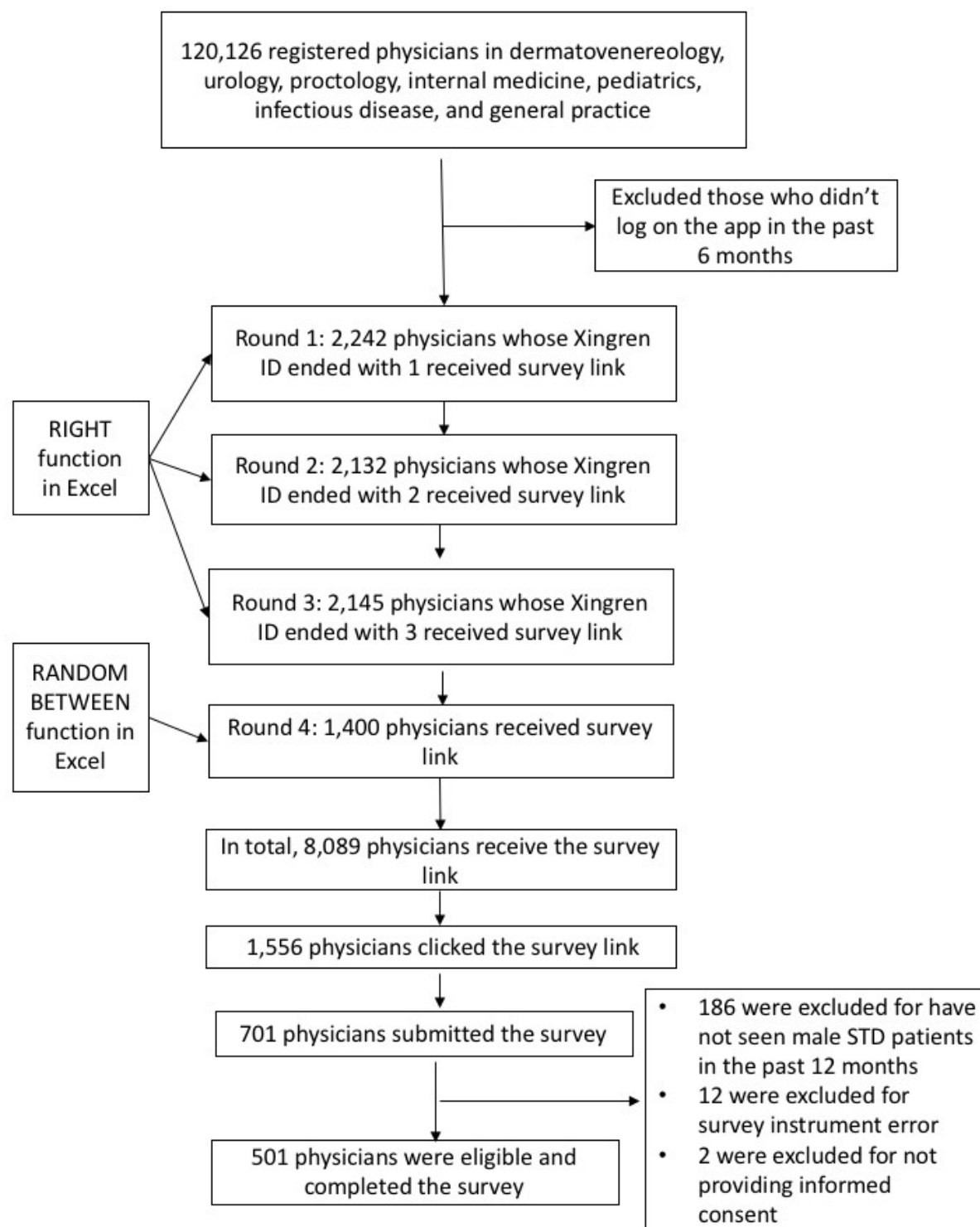

Supplement: Multimedia Appendix 1 [file mhealth_v6i10e10531_app1.pdf]
